# Development of an instructional movie illustrating a standardized clinical examination on patients with TMD symptoms

**DOI:** 10.3205/zma001332

**Published:** 2020-06-15

**Authors:** Angelika Rauch, Sebastian Florian Hahnel, Oliver Schierz

**Affiliations:** 1 University of Leipzig, Department of Prosthodontics and Materials Science, Leipzig, Germany

**Keywords:** instructional video, students, dental education, orofacial pain

## Abstract

**Objectives: **The aim of this project was to develop an instructional video that demonstrates a standardized clinical examination on patients with suspected temporomandibular disorders (TMD). After viewing the video, the learner should be knowledgeable about the examination steps and application of the examination techniques.

**Methods:** The instructional video was created by two dentists who are experienced in assessing patients with suspected TMD. Additionally, both examiners were calibrated according to the Diagnostic Criteria for Temporomandibular Disorders (DC/TMD). The instructional video was divided into chapters. Various camera angles, key points, image enlargements, and replays were used to better depict essential aspects of the assessment. Background noise was reduced to a minimum.

**Results: **The instructional video was modified and completed in two phases: the first by an experienced dentist and the second by a dentist specialized in TMD. The final video includes nine chapters and is 26.5 minutes in length (https://doi.org/10.5061/dryad.k8r7qc1).

**Conclusion: **Divided into chapters, this German instructional video shows an optimally timed, standardized clinical assessment of patients with suspected TMD.

## Zusammenfassung

**Zielsetzung:** Ziel dieser Arbeit war die Erstellung eines Instruktionsvideos, welches das Erlernen der Methodik zur Untersuchung von Patienten mit Verdacht auf craniomandibuläre Dysfunktionen (CMD) unterstützen soll. Nach Betrachten des Videos sollte der Lernende den Ablauf einer Untersuchung und die Anwendung der Untersuchungstechniken kennen.

**Methodik:** Ein Instruktionsvideo wurde von zwei Zahnärzten, welche in der Befunderhebung von Patienten mit Verdacht auf CMD erfahren sind, angefertigt. Zusätzlich waren beide Untersucher gemäß der Diagnostic Criteria for Temporomandibular Disorders (DC/TMD) kalibriert. Das Instruktionsvideo wurde in Kapiteln angelegt. Für eine verbesserte Darstellung wesentlicher Aspekte der Befunderhebung wurden verschiedene Kameraperspektiven, Stichpunkte, Bildvergrößerungen und Wiederholungen der Inhalte verwendet. Hintergrundgeräusche wurden auf ein Minimum reduziert.

**Ergebnisse: **Mittels zweier Evaluationsphasen, wobei die erste durch erfahrene und die zweite durch auf CMD-spezialisierte Zahnärzte erfolgte, wurde das Instruktionsvideo sukzessive modifiziert und fertiggestellt. Das finale Video umfasst neun Kapitel und hat eine Gesamtdauer von 26,5 Minuten (https://doi.org/10.5061/dryad.k8r7qc1).

**Schlussfolgerung: **Das deutsche Instruktionsvideo stellt den zeitlich optimierten Ablauf einer standardisierten Befunderhebung in Kapiteln dar, welche bei Patienten mit Verdacht auf craniomandibuläre Dysfunktion angewendet werden kann.

## 1. Introduction

Symptoms of temporomandibular disorders (TMD) include orofacial pain, sounds in the temporomandibular joint, and/or limited mobility of the mandible [1]. In the general population, it is presumed TMD has an estimated yearly incidence of 3.9% [2]. Data specifying the prevalence of TMD symptoms fluctuate depending on age, gender, and the individual reporting system [3] and, in Germany, are reported with numbers of up to 21.3% or 50.1% (according to patient history or clinical signs, respectively) [4]. Generally, a need for treatment is suspected in every sixth adult [5]. In addition to dentistry, TMD is known to influence various (chronic) diseases in other specialties [6]. Discussions are being held whether TMD is linked to headaches, neck pain, back pain, fibromyalgia, and even a variety of ear conditions [7], [8], [9], [10], [11]. In addition, psychosocial factors such as stress, coping mechanisms, depression, and the tendency to catastrophize play important roles in the etiopathogenesis of TMD [8].

Due to the multifactorial causes of TMD, it is essential to use both a valid and reliable measuring tool to correctly diagnose TMD. The Diagnostic Criteria for Temporomandibular Disorders (DC/TMD), published in 2014, meets these requirements and is even available in various languages [12], including an official German version that was published at the end of 2018 [13]. The DC/TMD includes a manual, which describes the verbal and practical elements of the exam, and diagnostic algorithms, that aim to standardize formulating diagnoses from the clinical assessment. 

Learning the process and technique of an assessment in patients with TMD using the manual provided by the DC/TMD is time-consuming and may cause users to make mistakes during the practical implementation. In general, difficulties in learning clinical skills are also known to occur with other (medical) examination techniques and treatment decisions. Instructional videos can help users learn, improve, and brush up on practical skills [14], [15], [16], [17], [18]. Videos that show the assessment of patients with suspected TMD according to the DC/TMD have been available in both Swedish and English for a few years [19], [20]. A German version has yet to be produced. 

The goal of this project was to create an instructional video that demonstrated a clinical examination of patients with suspected TMD in the German language.

## 2. Project description

To help train students as well as medical and dental professionals, a German-speaking instructional video should be created to demonstrate how to perform a structured and standardized TMD assessment according to the DC/TMD. Particular attention should be placed on how the examiner correctly uses verbal commands and examination techniques. The commands should strictly adhere to the German manual of the DC/TMD, while the patient case should follow the case reports in the English-speaking instructional video. In addition, common obstacles within the assessment should also be included. By watching the video, the following should be conveyed:

- The learner should know the steps to the exam according to the DC/TMD.
- The learner should know the exam techniques according to the DC/TMD.

The DC/TMD guidelines specify an exam timeframe of 20 minutes [21]. Since the general suggested length of a video ranges from six to nine minutes [22], the video should be divided into chapters according to the headings of the DC/TMD examination form (see table 1 (Tab. 1)). This also allows the learner to review the examination procedure successively. 

The introduction of the examination techniques should be filmed using a frontal view of the patient for a better initial overview of the procedure. In addition, a second camera perspective should be shown when measuring the “Opening and Closing Movements” to allow the viewer to better read off the numerical values in the sagittal plane. The palpation of the left half of the face, which was already performed on the contralateral side, should also be portrayed in two perspectives to give the viewer a more detailed understanding of the procedure. Important terms such as pain, familiar pain, familiar headache, and referred pain should be displayed and repeated during the examination. 

In the chapter “Incisal Relationships”, marking the reference points should be depicted in three successive, enlarged pictures alongside the running examination video. The fundamental principles of palpation and the areas of palpation should be added as key points in picture form at the beginning of the section “Muscle and TMJ Pain with Palpation”. Here, a picture of the patient from the video should be used. In contrast to the other sections, in the chapter “Muscle and TMJ Pain with Palpation” an additional picture showing the points of palpation should be displayed during the examination of the right side of the face, allowing the viewer to understand the corresponding points clearly. The clinical examination of the left side of the face should be depicted in two views. To better follow the palpation procedure in the section “Supplemental Muscle Pain with Palpation”, which includes the examination of the palpable intraoral muscles, the name of the respective muscle being palpated should be displayed.

In general, the calibration of the palpating finger should be visualized by displaying a scale indicating the necessary pressure. At the end of each section, a summary of the findings should be displayed, which are then virtually inserted into the correct areas of the DC/TMD examination form. Attention should also be paid to reduce the surrounding noise to a minimum.

## 3. Results

In 2016, video sequences were recorded showing the clinical examination on a patient according to the DC/ TMD guidelines. Both authors of the movie previously took part in DC/TMD trainings and calibration courses offered by the INfORM consortium to learn the examination criteria (AR Level 2, OS Level 3 of 3). In the following two years, the video was cut, and notes/illustrations were added to appropriate sections of the examination according to the project description. In February 2018, the first version of the video was shown to ten experienced dentists, evaluated according to the *Think-Aloud method*, and modified accordingly. In November 2018, the video was shown to and evaluated by ten participants of a DC/TMD training workshop and two DC/TMD gold standard examiners (Thomas List und Birgitta Häggman-Henrikson, Malmö University, Sweden) who led the training. Suggested changes were added to the video. In February 2019, the final version of the 26.5-minute German-language instructional video was completed (https://doi.org/10.5061/dryad.k8r7qc1). The nine sections (excluding introduction and credits) of the video vary in length from 0:46 to 8:19 minutes (see table 1 (Tab. 1)).

## 4. Discussion

The final instructional movie illustrates gathering clinical findings in a standardized patient with suspected TMD and can, therefore, be used as a learning tool for both students as well as medical and dental professionals. As a free online learning tool, which is available at DRYAD (https://doi.org/10.5061/dryad.k8r7qc1), it can be used in a classroom setting and during self-guided studying. This is particularly interesting, as results from a Swedish research group have shown that individual preparation for a clinical examination according to the DC/TMD using the manual and instructional video achieved CMD diagnoses with a similar degree of accuracy compared to examiners who previously participated in a practical training course [23]. Nevertheless, video-based learning should not be used as the sole method to learn the examination technique, but rather as a supplementary tool alongside classical instructor-based practical training. Especially for inexperienced learners, feedback from a supervisor is an integral part of correctly learning the examination skills [24]. For experienced users, an instructional video in terms of a* Just-in-time* training can function as a possible method to refresh and improve previously learned skills [18]. 

The completed instructional video fulfills various requirements that are necessary for effective use in teaching [25]. Important aspects of the assessment were highlighted using multiple camera perspectives, closeups, as well as relevant key points. Due to the length of an exam in patients with suspected TMD, the recommended time of an instructional video (9 minutes) could not be fulfilled [22]. Therefore, the video was cut into chapters according to the order of the examination. Disruptive factors, such as background music or noise, were reduced to a minimum or avoided. The rigid conversation structure defined by the DC/TMD manual can be seen as a limitation, causing the dialogue to appear less colloquial and sometimes rather monotonous. By adding a narrator to the video, viewers could have been addressed directly and additional explanations could have been inserted when necessary. Due to the extra length this would have added to the video, a narrator was not included. Furthermore, cultural differences in language (e.g., Swiss German or Austrian German) were not accounted for due to the lack of translations offered by the DC/TMD. 

## 5. Conclusions

This German instructional video gives the learner the opportunity to follow an optimally timed and standardized clinical assessment. It illustrates the proper examination techniques and verbal commands according to the DC/TDM manual. Due to its division into chapters, this video is also ideal for step-by-step learning or repetition of various examination techniques.

## Audiovisual material

The video is available from the Dryad Digital Repository: https://doi.org/10.5061/dryad.k8r7qc1 [26].

## Competing interests

The authors declare that they have no competing interests. 

## Figures and Tables

**Table 1:**
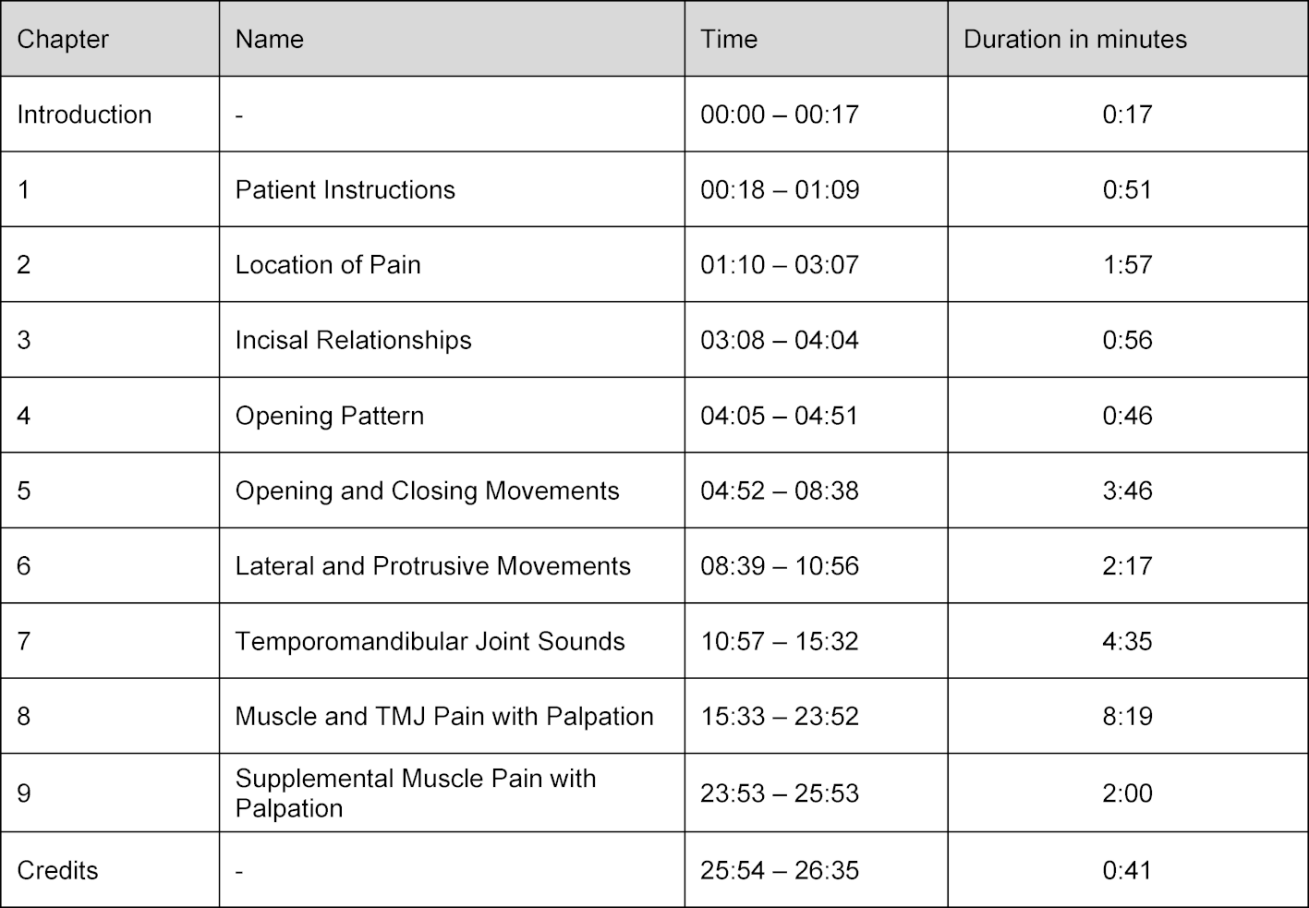
Timeline of the video

## References

[1] LeResche L (1997). Epidemiology of temporomandibular disorders: implications for the investigation of etiologic factors. Crit Rev Oral Biol Med.

[2] Slade GD, Ohrbach R, Greenspan JD, Fillingim RB, Bair E, Sanders AE, Dubner R, Diatchenko L, Meloto CB, Smith S, Maixner W (2016). Painful Temporomandibular Disorder: Decade of Discovery from OPPERA Studies. J Dent Res.

[3] Manfredini D, Guarda-Nardini L, Winocur E, Piccotti F, Ahlberg J, Lobbezoo F (2011). Research diagnostic criteria for temporomandibular disorders: a systematic review of axis I epidemiologic findings. Oral Surg Oral Med Oral Pathol Oral Radiol Endod.

[4] Micheelis W, Heinrich R (1999). Dritte Deutsche Mundgesundheitsstudie (DMS III): Ergebnisse, Trends und Problemanalysen auf der Grundlage bevölkerungsrepräsentativer Stichproben in Deutschland 1997.

[5] Al-Jundi MA, John MT, Setz JM, Szentpétery A, Kuss O (2008). Meta-analysis of treatment need for temporomandibular disorders in adult nonpatients. J Orofac Pain.

[6] Ohrbach R, Dworkin SF (2019). AAPT Diagnostic Criteria for Chronic Painful Temporomandibular Disorders. J Pain.

[7] Speciali JG, Dach F (2015). Temporomandibular dysfunction and headache disorder. Headache.

[8] List T, Jensen RH (2017). Temporomandibular disorders: Old ideas and new concepts. Cephalalgia.

[9] Ayouni I, Chebbi R, Hela Z, Dhidah M (2019). Comorbidity between fibromyalgia and temporomandibular disorders: a systematic review. Oral Surg Oral Med Oral Pathol Oral Radiol.

[10] Kusdra PM, Stechman-Neto J, Leão BL, Martins PF, Lacerda AB, Zeigelboim BS (2018). Relationship between Otological Symptoms and TMD. Int Tinnitus J.

[11] Skog C, Fjellner J, Ekberg E, Häggman-Henrikson B (2019). Tinnitus as a comorbidity to temporomandibular disorders-A systematic review. J Oral Rehabil.

[12] Schiffman E, Ohrbach R, Truelove E, Look J, Anderson G, Goulet JP, List T, Svensson P, Gonzalez Y, Lobbezoo F, Michelotti A, Brooks SL, Ceusters W, Drangsholt M, Ettlin D, Gaul C, Goldberg LJ, Haythornthwaite JA, Hollender L, Jensen R, John MT, de Laat A, de Leeuw R, Maixner W, van der Meulen M, Murray GM, Nixdorf DR, Palla S, Petersson A, Pionchon P, Smith B, Visscher CM, Zakrezewska J, Dworkin SF, International RDC/TMD Consortium Network, International association for Dental Research, Orofacial Pain Special Interest Group, International Association for the Study of Pain (2014). Diagnostic Criteria for Temporomandibular Disorders (DC/TMD) for Clinical and Research Applications: recommendations of the International RDC/TMD Consortium Network* and Orofacial Pain Special Interest †. J Oral Facial Pain H.

[13] Asendorf AE, Eberhard L, Daniel-Schierz S, Schierz O, Rammelsberg P, Giannakopoulos N (2018). Diagnostic Criteria for Temporomandibular Disorders:Assessment Instruments (German).

[14] Bäwert A, Holzinger A (2019). Practice makes perfect! Patient safety starts in medical school: Do instructional videos improve clinical skills and hygiene procedures in undergraduate medical students?. GMS J Med Educ.

[15] Reed S, Shell R, Kassis K, Tartaglia K, Wallihan R, Smith K, Hurtubise L, Martin B, Ledford C, Bradbury S, Bernstein HH, Mahan JD (2014). Applying adult learning practices in medical education. Curr Probl Pediatr Adolesc Health Care.

[16] Lee SC, Huang H, Minard CG, Schackman J, Rajagopalan S (2019). The use of podcast videos for airway skills. Clin Teach.

[17] Augestad KM, Butt K, Ignjatovic D, Keller DS, Kiran R (2019). Video-based coaching in surgical education: a systematic review and meta-analysis. Surg Endosc.

[18] Wang V, Cheng YT, Liu D (2016). Improving education: just-in-time splinting video. Clin Teach.

[19] Gonzalez Y, Chwirut J, List T, Ohrbach R (2014). DC/TMD Examination Protocol. MedEdPORTAL Publications.

[20] Ilgunas A, Harfeldt K, Alstergren P (2014). Diagnostic Criteria for Temporomandibular Disorders DC/TMD: Specialistversion. http://emmer.com/RDC/DC-TMD%20SWE%20Spec%20141103%20WEB.mp4.

[21] Alstergren P, Gonzalez-Stucker Y, Castrillon E, Peck CC, Goulet JP, Koutris M (2016). Guidlines for DC/TMD Training and Calibration. https://ubwp.buffalo.edu/rdc-tmdinternational/wp-content/uploads/sites/58/2017/01/DCTMD-Training-and-Calibration-160401.pdf.

[22] Guo PJ, Kim J, Rubin R (2014). How video production affects student engagement: an empirical study of MOOC videos.

[23] Österlund C, Berglund H, Akerman M, Nilsson E, Petersson H, Lam J, Alstergren P (2018). Diagnostic criteria for temporomandibular disorders: Diagnostic accuracy for general dentistry procedure without mandatory commands regarding myalgia, arthralgia and headache attributed to temporomandibular disorder. J Oral Rehabil.

[24] Karim J, Marwan Y, Dawas A, Esmaeel A, Snell L (2019). Learning knee arthrocentesis using YouTube videos. Clin Teach.

[25] Brame CJ (2016). Effective Educational Videos: Principles and Guidelines for Maximizing Student Learning from Video Content. CBE Life Sci Educ.

[26] Rauch A, Hahnel SF, Schierz O (2020). Data from: Development of an instructional movie illustrating a standardized clinical examination on patients with TMD symptoms.

